# Changes in knowledge and attitudes towards mental disorders in a Chinese sample population—a web-based approach

**DOI:** 10.1186/s12889-025-25644-2

**Published:** 2025-12-24

**Authors:** Juan Li, Yan-na Kou, Meng-meng Zhang, Jiao Dong, Meng-meng Li, Wei-li Yang, Yuening Li, Zhao-hui Zhang, Xiu-juan Wang

**Affiliations:** 1 The Second Affiliated Hospital of Xinxiang Medical University, Xinxiang, China; 2 Henan Collaborative Innovation Center of Prevention and treatment of mental disorder, Xinxiang, China; 3 Xinxiang Key Laboratory of Child and Adolescent Psychiatry, Xinxiang, China; 4 Henan Engineering Research Center of Physical Diagnostics and Treatment Technology for the Mental and Neurological disease, Xinxiang, China; 5 The First Affiliated Hospital of Xinxiang Medical University, Xinxiang, China; 6 Henan Key Laboratory of Neurorestoratology, Xinxiang, China

**Keywords:** Mental health knowledge, Stigma, Chinese general population

## Abstract

**Background:**

On October 10, 2022, The Lancet Psychiatry launched a global campaign aimed at ending the stigma surrounding mental illness, reinforcing the need for continued efforts to combat this pervasive issue. We previously surveyed knowledge and attitudes towards mental illness in a Chinese sample population. Seven years later, we aimed to conduct a study to reassess public perceptions of mental health and the level of mental illness literacy within the community today.

**Methods:**

A cross-sectional survey was designed and administered via a web-based chat application (WeChat) from March to September 2024. The survey tools were consistent with those employed in 2017. The Mental Health Knowledge Questionnaire (MHKQ) and the Perceived Devaluation and Discrimination Scale (PDDS) were used to evaluate the respondents’ mental health knowledge and attitudes towards mental illness. The study protocol was approved by the Research Ethics Committee of the Second Affiliated Hospital of Xinxiang Medical University.

**Results:**

A total of 1061 respondents were involved in the study. When education was included as a covariate, the adjusted mean total scores for the PDDS and MHKQ were (36.03 ± 0.19) and (16.51 ± 2.53), respectively, significantly higher than the survey results of 2017. Females were more closely associated with positive attitudes towards mental illness than males (*t* = − 2.06, *P* = 0.039). Respondents in the following groups were associated with higher MHKQ scores (all *P* < 0.05): Females; those with higher education levels; those who have had experience of others with mental illness; those who have learned about mental illness through personal experiences; those under 45 years old; and urban residents.

**Conclusions:**

Attitudes and knowledge levels showed positive changes between the two survey periods, suggesting potential improvements in mental health literacy among surveyed populations.More anti-stigma campaigns should be carried out for certain targeted groups, such as rural residents and middle-aged and elderly groups.

## Background

Mental illnesses, including depression, bipolar disorder, and schizophrenia, pose a significant public health challenge worldwide, with prevalence rates estimated to affect nearly one in four individuals at some point in their lives [1, 2]. In China, the weighted 12-month and lifetime prevalence rates of any mental illness (excluding dementia) are 9.3% and 16.6%, respectively [3]. Despite the increasing availability of effective treatment options, a considerable treatment gap remains [4]. One substantial barrier impeding individuals from seeking help is the pervasive stigma associated with mental illness [5, 6]. Many individuals fear discrimination and negative judgment from peers, family members, and society at large. This fear can lead to delays in seeking care, increased illness severity, and hesitation to adhere to prescribed treatment protocols, thereby exacerbating the impact of these disorders [7]. Second, the policies and resource allocation of healthcare institutions can also be influenced by discriminatory attitudes. In some regions, funding and resources for mental health services are often insufficient, leading to a decline in service quality. For example, in China, the management of medications and the provision of mental health services in primary healthcare institutions are restricted, resulting in many patients being unable to access timely and effective treatment [8].

The stigma surrounding mental illness can have detrimental impacts on mental health outcomes and quality of life [9], which can be worse than the impact of the mental health condition itself [10]. Delayed treatment due to shame or fear can result in worsening symptoms, increased functional impairment, and a higher likelihood of chronic mental health issues [7, 11]. Furthermore, stigma often contributes to non-adherence to medication regimens, leading to a cycle of relapse and recovery that is both taxing and demoralizing for patients [12]. For some, the compounded effects of stigma and untreated mental illness may culminate in severe situations such as depression and elevated suicide risk [13]. The urgency to manage and combat societal attitudes that perpetuate stigma is clear, as these negative perceptions can have a harmful impact on those living with mental health conditions.

Over the past decades, there has been a surge in the number of anti-stigma initiatives implemented on a global scale, aimed at alleviating the burden of prejudice and discrimination associated with mental illness [14]. These efforts, led by non-profit organizations, health organizations, and academic institutions, seek to educate the public, engage individuals with lived experiences, and promote narratives that highlight recovery and resilience. Strategies such as public campaigns, educational programs, and community movements have garnered notable success in challenging stigmatizing beliefs and promoting acceptance.

In China, Guangzhou also initiated the anti-stigma movement, mainly by training healthcare workers to enhance their knowledge of psychiatry, and this approach has improved discriminatory attitudes towards mental illness [15, 16]. In the past 5 years, China has launched nationwide activities such as “Healthy China initiative“(2019–2030) and “Science Popularization China” to promote health concepts and improve public health knowledge, which has significantly boosted mental health education and awareness. The dissemination of scientific knowledge through diverse channels, including educational videos and articles, has greatly enhanced public access to information about mental illness, which has led to a gradual transformation in societal attitudes. Social media platforms, in particular, have played a crucial role in encouraging open discussion about mental illness, facilitating a cultural shift that emphasizes understanding and support for mental health, rather than stigma or exclusion for those affected.

On October 10, 2022, The Lancet Psychiatry launched a global campaign aimed at ending the stigma surrounding mental illness, reinforcing the need for continued efforts to combat this pervasive issue [14]. Mental health education is one of the important strategies to improve the public’s attitude towards mental illness. Through systematic mental health education, it is possible to effectively reduce prejudice and discrimination against mental illness and enhance the public’s mental health literacy. The Healthy China initiative (2019–2030) encourages healthcare workers to carry out health popularization education in society, and our country has conducted many popular science initiatives regarding mental health. In 2017, we carried out a survey to examine knowledge and attitudes towards mental illness in a Chinse sample population [17], highlighting the need to strengthen health education in rural areas and among the elderly population. With the ongoing Healthy China Action, we would like to conduct another survey to reassess public perceptions of mental health and the level of mental illness knowledge within the community today. This study aims to shed light on the current status of attitudes towards mental illness and the levels of mental health literacy in the Chinese general population, so as to provide a foundational understanding that can guide future anti-stigma interventions and policy developments.

## Methods

### Participants

The questionnaire was administered using the Wenjuanxing platform, with all items set as mandatory fields to ensure data completeness. Participant recruitment employed convenience and snowball sampling methods, replicating the approach detailed in our previous study [17]. Specifically:

- Initial Seed Selection: We contacted 50 participants from the 2017 survey wave. These individuals were originally selected via convenience sampling based on their accessibility and willingness to participate (including researchers’ relatives and friends).
- Snowball Recruitment: These initial 2017 participants were asked to forward the survey link via WeChat to their own personal networks (like friends, family) and encouraged to share it further within their circles.

The instructions specified that anyone aged 18 or older could participate, and they could withdraw from the survey at any time. Rather than written informed consent, anonymous electronic informed consent was obtained from the respondents. The survey was conducted from March to September 2024. The study protocol was approved by the Research Ethics Committee of the Second Affiliated Hospital of Xinxiang Medical University (XYEFYLL-2024-44).

### Instruments

In line with our previous research [17], we collect basic demographic data, such as age, gender, education level, place of residence, and mental disorder history.

The Mental Health Knowledge Questionnaire (MHKQ) is a valuable tool designed to assess individuals’ knowledge of mental health issues. It comprises 20 self-administered items with scores ranging from 0 to 20, with higher scores indicating greater knowledge of mental health [18].

The Perceived Devaluation and Discrimination Scale (PDDS) is a widely used research tool designed to assess perceived devaluation and discrimination which may be experienced in various social contexts [19]. We used the Chinese version of PDD with a Cronbach’s alpha coefficient of 0.7 while the Cronbach’s alpha coefficient in this study is 0.63. The scale comprises 12 items, each rated on a five-point Likert scale, allowing respondents to indicate the extent of their agreement or disagreement with statements related to devaluation and discrimination. Scores can range from 12 to 60. Higher scores indicate lower levels of perceived discrimination.

### Data analysis

Data were analyzed using IBM SPSS v. 25. The statistical methods employed included a general description of the quantitative data. The normality of the data distribution was assessed using the Shapiro-Wilk test, and Levene’s test (α = 0.05) was employed to evaluate the homogeneity of variances between groups. The MHKQ total scores were normally distributed with homogeneity of variances(*F* = 1.229, *P* = 0.268), whereas the PDD total scores violated the homogeneity assumption༈*F* = 56.838, *P*<0.001༉. Therefore, an independent samples t-test was used for MHKQ, and Welch’s t-test was applied for PDD.The chi-square test is employed for categorical variables like sex and residence. For ordinal variables, the Mann-Whitney U test is employed. Group differences in the total scores of the PDDS and MHKQ were compared by covariance analysis with education as a covariate. The level of significance was set at 0.05.

## Results

### Sample characteristics

A total of 1061 respondents completed the questionnaires. The mean age of the respondents was (32.44 ± 9.49) years, with a range of 18–67. There were 378 (35.6%) male and 683 (64.6%) female respondents included in the study. The comparison of respondent characteristics between the two groups are summarized in Table 1.

**Table 1:** Comparison of respondent characteristics between 2017 and 2024

Variable		2017 (*n* = 1087, %)	2024 (*n* = 1061, %)	x^2^/t value	*P* value
Gender	Male	394 (36.2)	378 (35.6)	0.090	0.765
	Female	693 (63.8)	683 (64.6)		
Age (years)	16–24	114 (10.5)	110 (10.4)	5.625	0.131
	25–34	558 (51.3)	577 (54.4)		
	35–44	235 (21.6)	236 (22.2)		
	45 and above	180 (16.6)	138 (13.0)		
Education	Junior high school or less	97 (8.9)	70 (6.6)	9.081	0.028
	Senior high school	160 (14.7)	126 (11.9)		
	College degree/undergraduate	608 (55.9)	624 (58.8)		
	Postgraduate or above	222 (20.4)	241 (22.7)		
Place of residence	Urban area	901 (82.9)	863 (81.3)	0.879	0.348
	Rural area	186 (17.1)	198 (18.7)		
Contact level	Never	381 (35.1)	469 (44.2)	18.81	< 0.001
	Ever	706 (64.9)	592 (55.8)		
Diagnosed of mental disorder	Yes	59 (5.4)	56 (5.3)	0.02	0.877
	No	1028 (94.6)	1005 (94.7)		
Total score of PDDS		33.77 ± 6.66	36.02 ± 0.19	−5.55	< 0.001
Total score of MHKQ		15.89 ± 2.69	16.51 ± 5.52	−8.53	< 0.001
Sources of information about mental illness	Mass media	649 (59.7)	739 (69.7)	23.23	< 0.001
	Personal encounters	438 (40.3)	322 (30.3)		
Attitudes toward psychotropic drugs	Effective	727 (66.9)	749 (70.6)	7.20	0.066
	Get worse	40 (3.7)	27 (2.5)		
	Lead to dependency	280 (25.8)	261 (24.6)		

*Abbreviations*: *PDD* Perceived Devaluation and Discrimination, *MHKQ* Mental Health Knowledge Questionnaire, *P* *P*-value (two-tailed)

### Response frequencies for the additional items

For the question, “Do you ever have contact with people with mental illness?”, 44.2% (*n* = 469) replied “Never,” while 55.8% (*n* = 592) replied “Ever”. A total of 69.7% (*n* = 739) participants reported learning about mental illness from portrayals in mass media, while 30.3% (*n* = 322) reported learning from personal encounters with people who have mental illness. In regard to the respondents’ attitudes toward psychotropic drugs, 70.6% (*n* = 749) considered them effective, 2.5% (*n* = 27) believed that they would make patients worse, 24.6% (*n* = 261) believed that they are likely to lead to dependency, and 2.3% (*n* = 24) considered them ineffective (see Table 1).

### Comparison of the total scores of the PDDS and MHKQ between participants from 2017 to 2024

When education was included as a covariate, the adjusted mean total scores for the PDDS and MHKQ were (36.03 ± 0.19) and (16.51 ± 2.53), respectively. Compared with the data from the previous study, significant differences were found in both the MHKQ (*t*=−5.55, *P*<0.001, Cohen’s (d) = 0.24, 95%CI[0.152, 0.322]) and PDDS scores (Welch’s *t* =−8.53, *P*<0.001, Cohen’s (d)=−0.37, 95%CI[−0.45, −0.29]). The results are displayed in Table 1.

### Comparison of the results of the PDDS between participants from 2017 to 2024

The total scores of the PDDS ranged from 16 to 59, and the mean score was 36.02 (SD = 5.52). Compared with 2017, except for the item “Most people believe that attending a psychiatric hospital is a sign of personal failure” (F = 8.667, *P* = 0.07), significant differences were found in the other items (see Table 2).

**Table 2:** The results of the PDDS between participants from 2017 and 2024

Items	Years	Strongly agree (*n*, %)	Agree (*n*, %)	Unsure (*n*, %)	Disagree (*n*, %)	Strongly disagree (*n*, %)	Z value	*P*
1. Most people would accept a former mental patient as a close friend.	2017	49 (4.5)	149 (13.7)	545 (50.1)	253 (23.3)	91 (8.4)	−7.886	<0.001
	2024	55 (5.2)	194 (18.3)	624 (58.8)	154 (14.5)	34 (3.2)		
2. Most people believe that a person who has attended a mental hospital is just as intelligent as the average person.	2017	123 (11.3)	300 (27.6)	418 (38.5)	187 (17.2)	59 (5.4)	−3.481	<0.001
	2024	100 (9.4)	353 (33.3)	457 (43.1)	124 (11.7)	27 (2.5)		
3. Most people believe that a former mental patient is just as trustworthy as an average citizen.	2017	92 (8.5)	259 (23.8)	422 (38.8)	249 (22.9)	65 (6.0)	−3.420	0.001
	2024	86 (8.1%)	263 (24.8)	508 (47.9)	167 (15.7)	37 (3.5)		
4. Most people would accept a fully recovered former mental health patient as a teacher of young children in a public school.	2017	98 (9.0)	285 (26.2)	286 (26.3)	258 (23.7)	160 (14.7)	−2.896	0.004
	2024	92 (8.7)	263 (24.8)	408 (38.5)	194 (18.3)	104 (9.8)		
5. Most people believe that entering a mental hospital is a sign of personal failure.	2017	23 (2.1)	120 (11.0)	213 (19.6)	357 (32.8)	374 (34.4)	−0.414	0.679
	2024	26 (2.5)	91 (8.6)	246 (23.2)	317 (29.9)	381 (35.9)		
6. Most people would not hire a former mental health patient to take care of their children, even if he or she had been well for some time.	2017	360 (33.1)	421 (38.7)	169 (15.5)	96 (8.8)	41 (3.8)	−8.562	<0.001
	2024	197 (18.6)	431 (40.6)	290 (27.3)	99 (9.3)	44 (4.1)		
7. Most people think less of a person who has attended a mental hospital.	2017	116 (10.7)	397 (36.5)	256 (23.6)	233 (21.4)	85 (7.8)	−6.411	<0.001
	2024	46 (4.3)	293 (27.6)	390 (36.8)	241 (22.7)	91 (8.6)		
8. Most employers will hire a former mental health patient if he or she is qualified for the job.	2017	73 (6.7)	198 (18.2)	455 (41.9)	268 (24.7)	93 (8.6)	−3.624	<0.001
	2024	51 (4.8)	217 (20.5)	544 (51.3)	193 (18.2)	56 (5.3)		
9. Most employers will pass over the application of a former mental health patient in favor of another applicant.	2017	199 (18.3)	496 (45.5)	256 (23.6)	101 (9.3)	35 (3.2)	−6.011	<0.001
	2024	128 (12.1)	418 (39.4)	391 (36.9)	101 (9.5)	23 (2.2)		
10. Most people in my community would treat a former mental health patient the same as they treat anyone.	2017	83 (7.6)	311 (28.6)	440 (40.5)	209 (19.2)	44 (4.0)	−5.149	<0.001
	2024	88 (8.3)	343 (32.3)	498 (46.9)	119 (11.2)	13 (1.2)		
11. Most young women would be reluctant to date a man who has been hospitalized for serious mental illness.	2017	365 (33.6)	443 (40.8)	208 (19.1)	53 (4.9)	18 (1.7)	−12.029	<0.001
	2024	172 (16.2)	419 (39.5)	380 (35.8)	70 (6.6)	20 (1.9)		
12. Once they are aware that a person was attended a mental hospital, most people will take his or her opinions less seriously.	2017	124 (11.4)	430 (39.6)	342 (31.5)	162 (14.9)	29 (2.7)	−6.751	<0.001
	2024	60 (5.7)	329 (31.0)	471 (44.4)	172 (16.2)	29 (2.7)		

*Abbreviations:* *PDD *Perceived Devaluation and Discrimination

### Comparison of the results of the MHKQ between participants from 2017 to 2024

The total scores of the MHKQ ranged from 2 to 20 and the mean score was 16.51 (SD = 2.53). The mean score for the first section of true or false questions was 13.76 (SD = 1.99), and 181 (17.1%) respondents had perfect scores. Compared with 2017, the current study found significant differences in items 1, 2, 4, 5, 6, 11, and 14 for the first part. In respect to the last four questions, 436 (41.09%) respondents were aware of all four mental health promotion days. Compared with 2017, the public’s awareness rate of International Mental Health Day, International Suicide Prevention Day, and World Sleep Day has increased significantly (all *P* < 0.05) (Table 3).

**Table 3:** Comparison of the correct response rate of the MHKQ between participants from 2017 and 2024 (n, %)

Item	2017 (*n* = 1087)	2024 (*n* = 1061)	x^2^ value	*P* value	OR(95%CI)
1.Mental health is a component of health (true)	1047 (96.3)	1050 (99.0)	16.18	< 0.001	0.274(0.014–0.537)
2.Mental illness is caused by incorrect thinking. (false)	596 (54.8)	719 (67.8)	37.85	< 0.001	1.732(1.453–2.064)
3.Many people have mental health problems but do not realize it. (true)	1052 (96.8)	1031 (97.2)	0.28	0.596	0.875(0.533–1.435)
4.All mental illnesses are caused by external stressors. (false)	653 (60.1)	708 (66.7)	10.25	0.001	1.333(1.118–1.590)
5.Components of mental health include normal intelligence, stable mood, a positive attitude, quality interpersonal relationships, and adaptability. (true)	1035 (95.2)	1033 (97.4)	6.89	0.009	0.540(0.338–0.861)
6.Most mental illnesses cannot be cures. (false)	742 (68.3)	795 (74.9)	11.73	< 0.001	1.390(1.151–1.678)
7.Psychological or psychiatric services should be sought if one suspects the presence of psychological problems or a mental disorder. (true)	969 (89.1)	956 (90.1)	0.53	0.466	0.902(0.683–1.191)
8.Psychological problems can occur at almost any age. (true)	1049 (96.5)	1026 (96.7)	0.06	0.801	0.942(0.590–1.502)
9.Mental illness and psychological problems cannot be prevented. (false)	825 (75.9)	828 (78.0)	1.39	0.238	1.129(0.923–1.380)
10.Even for severe mental illness (e.g., schizophrenia), medications should be taken for a given period of time only; there is no need to take them for a long time. (false)	887 (81.6)	837 (78.9)	2.49	0.114	0.843(0.681–1.042)
11.Positive attitudes, good interpersonal relationships, and healthy life styles can help maintain mental health. (true)	1046 (96.2)	1039 (97.9)	5.44	0.020	0.540(0.320–0.913)
12.Individuals with a family history of mental illness are at a higher risk of psychological problems and mental illness. (true)	981 (90.2)	948 (89.3)	0.47	0.491	1.103(0.834–1.459)
13.Psychological problems in adolescents do not influence academic grades. (false)	964 (88.7)	939 (88.5)	0.02	0.894	0.982(0.753–1.281)
14.Middle-aged or elderly individuals are unlikely to develop psychological problems and mental illness. (false)	957 (88.0)	893 (84.2)	6.75	0.009	0.722(0.564–0.924)
15.Individuals with a poor temperament are more likely to have mental health problems. (true)	838 (77.1)	788 (74.3)	2.33	0.127	1.166(0.957–1.420)
16.Mental problems or disorders may occur when an individual is under psychological stress due to major life events (e.g., death of family members). (true)	1031 (94.8)	1006 (94.8)	0.001	0.973	1.007(0.687–1.475)
17.Have you heard about International Mental Health Day? (yes)	613 (56.4)	735 (69.3)	38.11	< 0.001	0.574(0.480–0.685)
18.Have you heard about the International Day against Drug Abuse and Illicit Drug Trafficking? (yes)	947 (87.1)	949 (89.4)	2.80	0.094	0.798(0.613–1.040)
19.Have you heard about International Suicide Prevention Day? (yes)	345 (31.7)	522 (49.2)	68.00	< 0.001	0.480(0.403–0.572)
20.Have you heard about World Sleep Day? (yes)	694 (63.8)	720 (67.9)	3.85	0.05	0.836(0.700–1.000.700.000)

*Abbreviations*: *MHKQ *Mental Health Knowledge Questionnaire

### Comparison of the PDDS among demographic variables in 2024

Table 4 shows the mean PDDS scores for different demographic groups. Statistically significant differences were found in gender-matched scores, with females scoring higher than males (*F* = − 2.06, *P* = 0.039).

**Table 4:** Comparison of the PDDS scores among the demographic variables between participants from 2017 and 2024

Item	2017 (*n* = 1087)	t/F value	*P* value	2024 (*n* = 1061)	t/F value	*P* value
Gender		0.67	0.500		−2.06	0.039
Male	33.96 ± 7.24			35.55 ± 4.95		
Female	33.66 ± 6.31			36.28 ± 5.80		
Age (years)		5.37	0.001		0.75	0.524
16–24	35.11 ± 5.48			35.97 ± 4.97		
25–34	34.03 ± 6.69			36.18 ± 5.31		
35–44	32.37 ± 7.11			35.56 ± 6.19		
45 and above	33.92 ± 6.39			36.17 ± 5.62		
Education		0.13	0.942		0.98	0.401
Junior high school or less	33.90 ± 6.76			37.04 ± 5.46		
Senior high school	33.82 ± 6.77			35.67 ± 5.17		
College/undergraduate	33.83 ± 6.58			35.99 ± 5.61		
Postgraduate or above	33.52 ± 6.79			35.99 ± 5.49		
Place of residence		−2.42	0.016		−1.67	0.095
Urban area	33.56 ± 6.74			35.88 ± 5.65		
Rural area	34.78 ± 6.20			36.61 ± 4.91		
Sources of information about mental illness		1.31	0.192		0.54	0.592
Mass media	33.99 ± 6.44			36.08 ± 5.40		
Personal encounters	33.44 ± 6.97			35.88 ± 5.81		
Contact level		1.40	0.161		0.19	0.852
Never	34.15 ± 6.54			36.06 ± 5.16		
Ever	33.56 ± 6.72			35.99 ± 5.80		

*Abbreviations*: *PDD* Perceived Devaluation and Discrimination

### Comparison of the MHKQ results among demographic variables in 2024

Table 5 shows the mean MHKQ scores for different demographic groups. Compared with those variables, the respondents’ gender, age, education levels, area of residence, sources of information about mental illness, and contact levels with people with mental illness were found to be significantly related to the total MHKQ scores. The analysis of variance showed that females scored significantly higher than males on the MHKQ (*t* = − 4.95, *P* = 0.001). Respondents aged 16–44 scored significantly higher than those above 45 years old on the MHKQ (*F* = 4.49, *P* = 0.04). Respondents with higher education levels were found to have greater mental health literacy (*F* = 34.49, *P* < 0.001). Urban residents had higher MHKQ scores than rural residents (*t* = 4.07, *P* < 0.001). Those who had prior experience of others with mental illness (*t* = − 4.54, *P* < 0.001) and who had learned about mental illness through personal experiences (*t* = − 3.05, *P* = 0.002) had higher MHKQ scores.

**Table 5:** Comparison of the MHKQ scores among the demographic variables between participants from 2017 and 2024

Item	2017	t/F value	*P* value	2024	t/F value	*P* value
Gender		−1.02	0.310		−4.95	0.001
Male	15.78 ± 2.80			16.00 ± 2.73		
Female	15.95 ± 2.63			16.80 ± 2.37		
Age (years)		4.56	0.003		4.49	0.004
16–24	15.82 ± 2.16			16.63 ± 2.73		
25–34	15.93 ± 2.86			16.69 ± 2.54		
35–44	16.27 ± 2.52			16.44 ± 2.40		
45 and above	15.30 ± 2.61			15.83 ± 2.45		
Education		65.72	< 0.001		34.49	< 0.001
Junior high school or less	13.36 ± 2.28			14.11 ± 2.67		
Senior high school	14.69 ± 2.36			16.05 ± 2.75		
College/undergraduate	16.17 ± 2.51			16.55 ± 2.39		
Postgraduate or above	17.08 ± 2.58			17.37 ± 2.23		
Place of residence		7.32	< 0.001		4.07	< 0.001
Urban area	16.15 ± 2.62			16.67 ± 2.48		
Rural area	14.60 ± 2.65			15.86 ± 2.64		
Sources of information about mental illness		−2.10	0.036		−3.05	0.002
Mass media	15.74 ± 2.54			16.36 ± 2.42		
Personal encounters	16.10 ± 2.89			16.87 ± 2.73		
Contact level		−4.85	< 0.001		−4.54	< 0.001
Never	15.35 ± 2.56			16.02 ± 2.54		
Ever	16.18 ± 2.72			16.76 ± 2.27		

*Abbreviations*: *MHKQ* Mental Health Knowledge Questionnaire

## Discussion

Our study indicates potential changes in knowledge of mental health and attitudes toward mental illness among participant compared to data from 7 years ago. However, given the cross-sectional convenience sampling method, these observed differences may primarily reflect variations in sample composition over time rather than definitive societal-level improvements. Since 2019, China has implemented the Healthy China Initiative (2019–2030), which incorporates health promotion efforts, such as requiring healthcare workers to engage in popular science work for career advancement. Many doctors have subsequently participated in community outreach or online health education. Concurrently, media portrayals of mental illness appear to have become more positive. In our sample, these external factors were possibly associated with shifts in knowledge and attitudes, but the study design didn’t directly assess participants’ exposure to or the impact of these initiatives, limiting causal inferences.

Moreover, we all know that the media plays a crucial role in shaping public attitudes towards mental illness. In the past, media coverage often tended to associate mental illness with negative images such as violence and crime, and these stereotypes reinforced the public’s fear and prejudice towards individuals with mental health issues [20]. However, with the advancement of the Healthy China initiative, more and more healthcare workers, including mental health professionals, are beginning to use short videos for public education. This may have contributed to broader awareness in our sample, which implies that professional involvement in mental health advocacy could be valuable for education efforts.Overall, our findings highlight the importance of continued efforts to enhance mental health literacy. Based on the sample data, improving understanding of mental health might encourage individuals to seek psychological assistance when needed. However, further research with representative samples is necessary to generalize these insights to the broader population.

In our detailed comparison using the PDDS, we found that, apart from the item “Most people believe that entering a mental hospital is a sign of personal failure,” which showed no significant differences between groups, all other items revealed notable intergroup differences. This indicates that discrimination against mental health facilities may persist in the study participants, potentially dissuading individuals from seeking early treatment.

More than 6 decades ago, many countries in Europe and America began the process of de-institutionalization, shifting away from traditional mental health hospitals [21]. In China, there have been significant efforts to rename psychiatric hospitals (e.g., changing the name of psychiatric hospitals to alternatives such as “brain hospital,” Anding Hospital or Kangning Hospital, etc.) or to transform these hospitals into general hospitals. The health administration of China requires general hospitals to set up departments of psychiatry, a decision which might prove instrumental in diminishing the stigma associated with visiting mental healthcare facilities.

In addition, closer examination of the MHKQ within our sample revealed increased awareness of important dates such as International Mental Health Day, International Suicide Prevention Day, and World Sleep Day compared to previous data. These findings may indicate the potential influence of health education initiatives. Notably, awareness of the International Day against Drug Abuse and Illicit Drug Trafficking approached 90% in our participants. However, it is evident that other mental health-related observances, particularly International Suicide Prevention Day—showed lower recognition rates in this sample, would benefit from stronger promotional efforts to bolster public awareness.

Further analysis of demographic differences within our sample revealed that female participants tend to exhibit greater acceptance and understanding in attitudes towards mental illness. This finding is in agreement with previous research [15, 22, 23] suggesting that societal expectations often encourage female to display empathy and compassion.

Consequently, female participants in our sample may be more inclined to adopt supportive and understanding attitudes when confronted with the struggles of others, which explains why females exhibit higher levels of tolerance towards mental health issues [24].

Furthermore, our results indicated higher levels of mental health knowledge among female respondents compared to males, consistent with earlier studies highlighting gender disparities in mental health awareness [15, 25]. Notably, in both surveys, the proportion of female respondents was higher. This may reflect a greater engagement with health-related topics or a higher willingness to participate in such research among the female in our sample, rather than a general population trend.To address this sampling imbalance, it could be beneficial for future studies to implement strategies aimed at increasing male participation, such as targeted outreach, tailored communication, or incentivized engagement.

In addition, within our sample, we found greater mental health literacy among urban populations, people under 45 years old, and individuals with higher education levels. This observation aligns with prior research [17, 26, 27], which suggests that urban residents have greater access to education compared to their rural counterparts. In the study population, higher education levels foster more opportunities for individuals to engage with mental health resources and education. Therefore, there is a pressing need to prioritize mental health education, particularly in rural areas where knowledge gaps may still exist.

Finally, it is essential to acknowledge the limitations of this study. First and foremost, this research was not a cohort study; therefore, the findings may not reflect long-term trends. In addition, the study used self-reported instruments which can be subject to social desirability bias and may not always accurately reflect participants’ true feelings or behaviors. Moreover, convenience sampling limits the sample’s representativeness. This is because the final sample depends on the social networks of the initially selected participants, which restricts generalizability. Additionally, the use of an online questionnaire led to demographic imbalance, with females and younger individuals overrepresented. Future research employing probabilistic sampling could more accurately describe attitude distributions across sociodemographic groups. Lastly, we used an electronic version of informed consent, meaning that it was not possible to ensure that every participant was fully clear about the research purposes; however, the participants completed the questionnaire voluntarily and were aware of their right to withdraw from the study at any time.

## Conclusions

In conclusion, our survey results observed positive differences in attitudes and knowledge levels within the specific groups studied between the 2017 and 2024. These differences suggest a potential change in mental health literacy among these surveyed groups. However, because the study used a cross-sectional convenience sampling design, the differences may reflect variations in sample composition rather than definitive societal-level trends over time. Sustained efforts focused on education and advocacy remain crucial, aiming to reduce stigma and support mental health literacy across diverse demographics. Future initiatives could explore incorporating engaging modalities like drama and performances into public education. This approach may enhance the reach and effectiveness of activities aimed at populations such as those in rural areas or older adults. Further research with representative samples and longitudinal designs is needed to assess population-level trends and causal links.

## Data Availability

All the data supporting our findings have been presented in the manuscript; however, the datasets analyzed during the current study are available from the corresponding author on reasonable request.

## References

[1] Rehm J, Shield KD. Global burden of disease and the impact of mental and addictive disorders. Curr Psychiatry Rep. 2019;21(2):10. 30729322 10.1007/s11920-019-0997-0

[2] Charlson F, van Ommeren M, Flaxman A, Cornett J, Whiteford H, Saxena S. New WHO prevalence estimates of mental disorders in conflict settings: a systematic review and meta-analysis. Lancet. 2019;394(10194):240–8. 31200992 10.1016/S0140-6736(19)30934-1 PMC6657025

[3] Huang Y, Wang Y, Wang H, Liu Z, Yu X, Yan J, et al. Prevalence of mental disorders in China: a cross-sectional epidemiological study. Lancet Psychiatry. 2019;6(3):211–24. 30792114 10.1016/S2215-0366(18)30511-X

[4] Mojiminiyi IO, Balogun MR, Ogunnowo BE. Knowledge and attitude towards mental disorders among adults in an urban community in south-west Nigeria. Malawi Med J. 2020;32(2):87–94. 35140845 10.4314/mmj.v32i2.6 PMC8788587

[5] Evans L, Chang A, Dehon J, Streb M, Bruce M, Clark E, et al. The relationships between perceived mental illness prevalence, mental illness stigma, and attitudes toward help-seeking. Curr Psychol. 2023. 10.1007/s12144-023-04329-2. 10.1007/s12144-023-04329-2 PMC9975862 37359578

[6] Ibrahim N, Amit N, Shahar S, Wee LH, Ismail R, Khairuddin R, et al. Do depression literacy, mental illness beliefs and stigma influence mental health help-seeking attitude? A cross-sectional study of secondary school and university students from B40 households in Malaysia. BMC Public Health. 2019;19(Suppl 4):544. 31196033 10.1186/s12889-019-6862-6 PMC6565530

[7] Chukwuma OV, Ezeani EI, Fatoye EO, Benjamin J, Okobi OE, Nwume CG, et al. A systematic review of the effect of stigmatization on psychiatric illness outcomes. Cureus J Med Sci. 2024;16(6):e62642. 10.7759/cureus.62642 PMC11258934 39036187

[8] Huang Y, Yao D, Xi X, Wang Y, Yao W. Current status of pharmacy services in primary healthcare institutions in Jiangsu Province, China. Aust J Prim Health. 2020;26(5):424–30. 32900425 10.1071/PY20038

[9] Anagnostouli M, Katsavos S, Artemiadis A, Zacharis M, Argyrou P, Theotoka I, et al. Determinants of stigma in a cohort of Hellenic patients suffering from multiple sclerosis: a cross-sectional study. BMC Neurol. 2016;16:101. 27411373 10.1186/s12883-016-0621-4 PMC4944520

[10] Thornicroft G, Sunkel C, Milenova M. How to stop stigma: implementing the lancet commission on ending stigma and discrimination in mental health. Lancet Psychiat. 2024;11(2):88–9. 10.1016/S2215-0366(23)00374-7 38008101

[11] Oexle N, Muller M, Kawohl W, Xu Z, Viering S, Wyss C, et al. Self-stigma as a barrier to recovery: a longitudinal study. Eur Arch Psychiatry Clin Neurosci. 2018;268(2):209–12. 28188369 10.1007/s00406-017-0773-2

[12] Abdisa E, Fekadu G, Girma S, Shibiru T, Tilahun T, Mohamed H, et al. Self-stigma and medication adherence among patients with mental illness treated at Jimma university medical Center, Southwest Ethiopia. Int J Ment Health Syst. 2020;14:56. 32760443 10.1186/s13033-020-00391-6 PMC7391813

[13] Wastler H, Lucksted A, Phalen P, Drapalski A. Internalized stigma, sense of belonging, and suicidal ideation among veterans with serious mental illness. Psychiatr Rehabil J. 2020;43(2):91–6. 31414842 10.1037/prj0000386 PMC7021557

[14] Thornicroft G, Sunkel C, Alikhon AA, Baker S, Brohan E, El CR, Davies K, Demissie M, Duncan J, Fekadu W, et al. The lancet commission on ending stigma and discrimination in mental health. Lancet. 2022;400(10361):1438–80. 36223799 10.1016/S0140-6736(22)01470-2

[15] Li J, Li J, Thornicroft G, Huang Y. Levels of stigma among community mental health staff in Guangzhou, China. BMC Psychiatry. 2014;14:231. 25115221 10.1186/s12888-014-0231-x PMC4149249

[16] Li J, Fan Y, Zhong HQ, Duan XL, Chen W, Evans-Lacko S, et al. Effectiveness of an anti-stigma training on improving attitudes and decreasing discrimination towards people with mental disorders among care assistant workers in Guangzhou, China. Int J Ment Health Syst. 2019;13:1. 30622627 10.1186/s13033-018-0259-2 PMC6317233

[17] Li J, Zhang MM, Zhao L, Li WQ, Mu JL, Zhang ZH. Evaluation of attitudes and knowledge toward mental disorders in a sample of the Chinese population using a web-based approach. BMC Psychiatry. 2018;18(1):367. 30453932 10.1186/s12888-018-1949-7 PMC6245628

[18] Cheng S, An D, Yao Z, Liu JJ, Ning X, Wong JP, et al. Association between mental health knowledge level and depressive symptoms among Chinese college students. Int J Environ Res Public Health. 2021. 10.3390/ijerph18041850. 33672872 10.3390/ijerph18041850 PMC7918134

[19] Yin HF, Xu GM, Yang GF, Tian HJ. Reliability and validity of the Chineseversion of the perceived D evaluation-discrimination scale in community population. Chin Ment Health J. 2014;28(1):63–9.

[20] Patel AD, Pal A, Rahat F, Yadav R, Tiwari P, Alam Z. Attitude towards patients with psychiatric illness among undergraduate medical students at government medical college: a cross-sectional study. J Fam Med Prim Care. 2023;12(4):756–61. 10.4103/jfmpc.jfmpc_2382_22 PMC10259568 37312798

[21] Lesage AD, Morissette R, Fortier L, Reinharz D, Contandriopoulos AP. Downsizing psychiatric hospitals: needs for care and services of current and discharged long-stay inpatients. Can J Psychiatry. 2000;45(6):526–32. 10986569 10.1177/070674370004500602

[22] Pang S, Liu J, Mahesh M, Chua BY, Shahwan S, Lee SP, Vaingankar JA, Abdin E, Fung D, Chong SA, et al. Stigma among Singaporean youth: a cross-sectional study on adolescent attitudes towards serious mental illness and social tolerance in a multiethnic population. BMJ OPEN. 2017;7(10):e16432. 10.1136/bmjopen-2017-016432 PMC5652546 29042379

[23] Wong EC, Collins RL, Cerully JL, Yu JW, Seelam R. Effects of contact-based mental illness stigma reduction programs: age, gender, and Asian, Latino, and white American differences. Soc Psychiatry Psychiatr Epidemiol. 2018;53(3):299–308. 29196773 10.1007/s00127-017-1459-9

[24] Hogberg T, Magnusson A, Lutzen K, Ewalds-Kvist B. Swedish attitudes towards persons with mental illness. Nord J Psychiatry. 2012;66(2):86–96. 21958390 10.3109/08039488.2011.596947

[25] Rong Y, Glozier N, Luscombe GM, Davenport TA, Huang Y, Hickie IB. Improving knowledge and attitudes towards depression: a controlled trial among Chinese medical students. BMC Psychiatry. 2011;11:36. 21385432 10.1186/1471-244X-11-36 PMC3062591

[26] Chen M, Lin GR, Wang GY, Yang L, Lyu N, Qian C, et al. Stigma toward mental disorders and associated factors among community mental health workers in Wuhan, China. Asia Pac Psychiatry. 2023;15(2–3):e12542. 37517868 10.1111/appy.12542

[27] Girma E, Tesfaye M, Froeschl G, Moller-Leimkuhler AM, Muller N, Dehning S. Public stigma against people with mental illness in the Gilgel Gibe field research center (GGFRC) in Southwest Ethiopia. PLoS One. 2013;8(12):e82116. 24324756 10.1371/journal.pone.0082116 PMC3853185

